# Supplementary material for: Delineating between-subject heterogeneity in alpha networks with Spatio-Spectral Eigenmodes
Source: Neuroimage. 2021 Oct 15;240:118330. doi: 10.1016/j.neuroimage.2021.118330 (PMC8456753; doi:10.1016/j.neuroimage.2021.118330)
Supplement: Supplementary Data S1 — Supplementary Raw Research Data. This is open data under the CC BY license http://creativecommons.org/licenses/by/4.0/ [file mmc1.pdf]

Appendix D. Individual realisation results for group simulation

In Figure D.10 we present the results for each of the 20 individuals in the simulation. These results are analogous to those shown for the example participant in panel F of Figure 1.

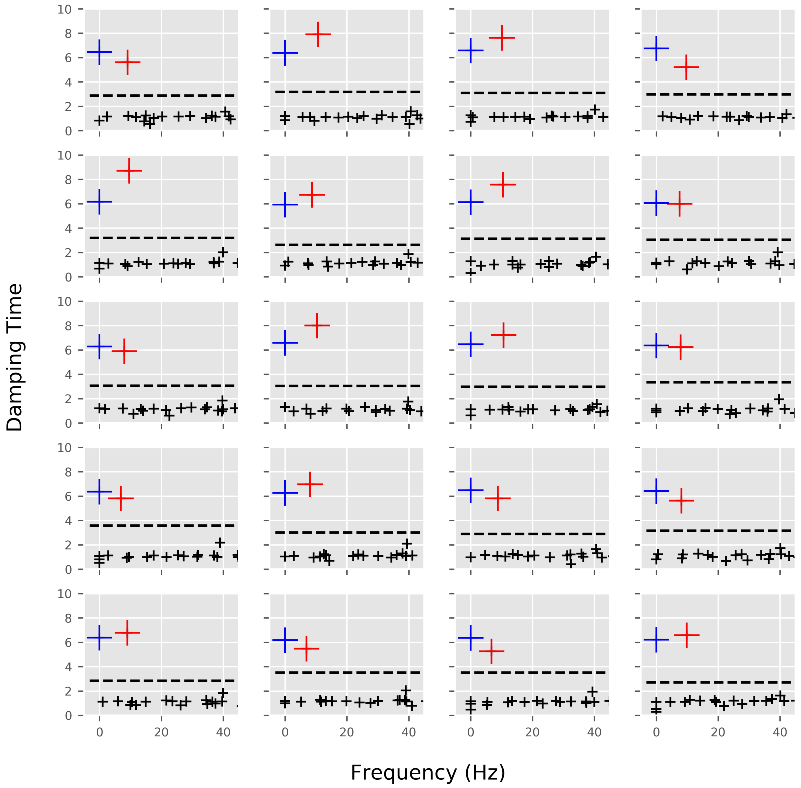

Figure D.10: Replication of figure 1F for all 20 individual simulation realisations. Damping-time of each mode as a function of frequency. The slope (blue cross) and 9Hz peak (red crosses) have the longest damping times indicating that the dynamics in these modes are relatively un-damped and possibly of greater dynamical importance to the system. The dashed line indicates the 99% significance threshold computed for each individual run via the permutation scheme described in the main text.

Appendix E. Noise varying simulation

The simulation from Figure 1 was repeated with a wide range of noise levels to describe the effect of noise on the mode identification. 12 levels of white noise with a standard-deviation ranging between 0.2 and 5 was added to the simulated system before model fitting and modal decomposition (Figure E.11A) The simulation analysis in the main text used white noise with a standard deviation of 1. The Fourier power spectra for a single node containing all three

resonances shows the effect of additional noise. All three resonances are clearly visible at low noise levels but the spectrum shows substantial flattening at very high noise levels (Figure E.11B). The eigenvalues of the modal decomposition show a similar response to additional noise. The peak frequencies are relatively consistent across noise levels though the damping time of the three main resonances reduces substantially at high noise levels (Figure E.11C). Critically, though greatly reduced, the damping times of the oscillatory signals remain above the noise floor even for the largest noise condition.

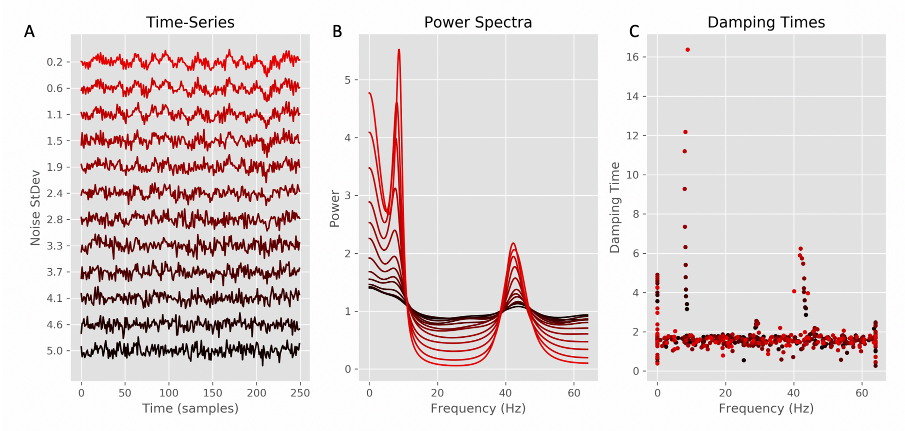

Figure E.11: Supplemental simulation containing varying noise levels.
A: A time-series segment for each of the noise levels.
B: Fourier power spectra for each of the noise levels - line colours match those in panel A
C: The modal damping times and peak frequencies for each of the noise levels - scatter point colours match those in panel A

Appendix F. Three mode simulation

A simulation with an third oscillatory mode was run to explore whether the model is able to represent modes with a wide spread in peak frequencies. The simulation scheme was identical to the one used for Figure 1 with an except that a third mode with peak frequency of 42Hz was added to each odd-numbered node in the network. 300 seconds of data were generated at 128Hz and an order-7 MVAR model used to describe any oscillatory dynamics. The modal decomposition was applied to the fitted MVAR parameters.

The Fourier power spectrum shows the three resonances in the system (Figure F.12A). These resonances are, in turn, well described by the largest modes in the modal decomposition (Figure F.12B). The distribution of modal eigenvalues shows that most of the modes are evenly spread across frequency and have short damping times (Figure F.12C and D). In contrast, the modes with

long damping times match the frequency of the simulated resonances at 0, 9 and 42Hz. Finally, the cross-spectra of the three oscillatory modes clearly identify the network structure of each resonance (Figure F.12E).

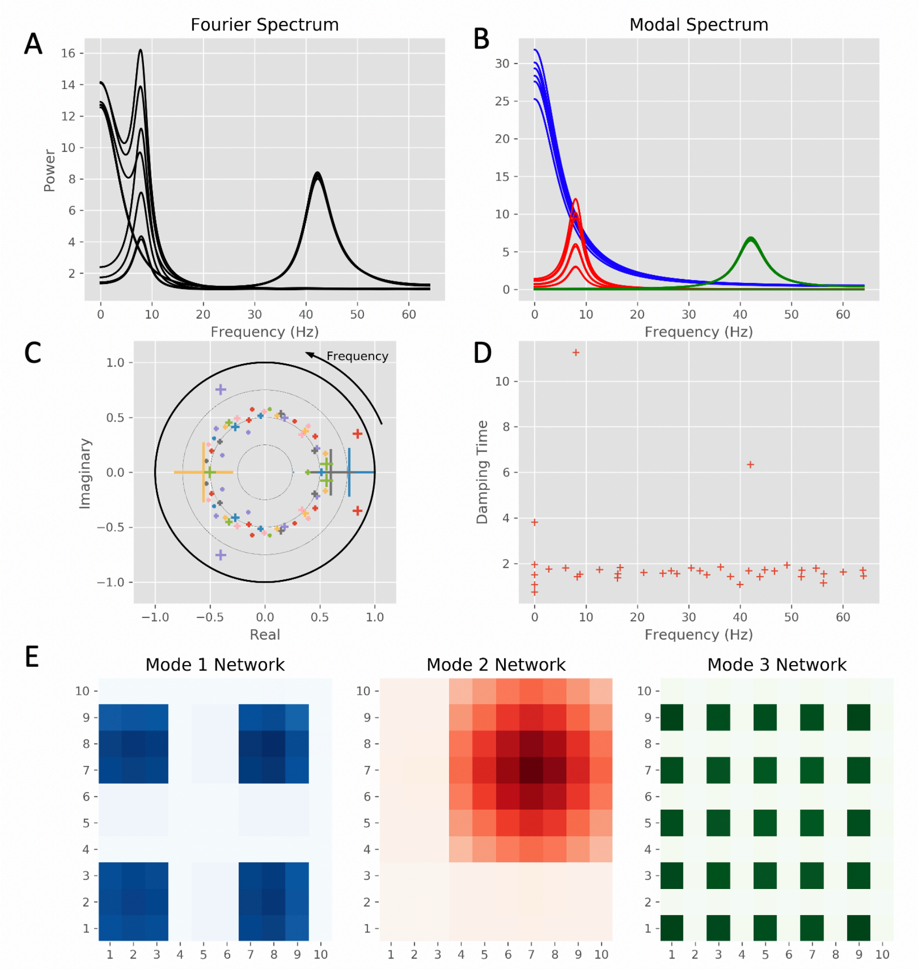

Figure F.12: Supplemental simulation with three oscillatory modes spread across a ten node network.
A: Fourier power spectra for each of the ten nodes.
B: Modal power spectra for each of the three resonances across the ten nodes.
C: The modal pole plot.
D: The damping time and peak frequency for each identified mode.
E: Network structure for the three modes. Colours match the spectra in panel B

Appendix G. Non-sinusoidal simulation

The MVAR model and SSE decomposition can give misleading results in the presence of strongly non-sinusoidal oscillations. Like all Fourier based analyses, a non-sinusiodal oscillation will induce a harmonic peak in the MVAR power spectrum which is, in turn, represented by a distinct oscillatory mode. Such harmonic modes are not easily distinguished from separate oscillations using the present methodology. Analysis of highly non-linear signals might be better suited to methods such as the Empirical Mode Decomposition (EMD) [76, 77] which is able to represent such non-linearities with dynamics in instantaneous frequency rather than harmonics. The MVAR-SSE method is still the appropriate choice for exploratory analysis of multivariate signals as it has the advantage of being fully multivariate, linear and deterministic.

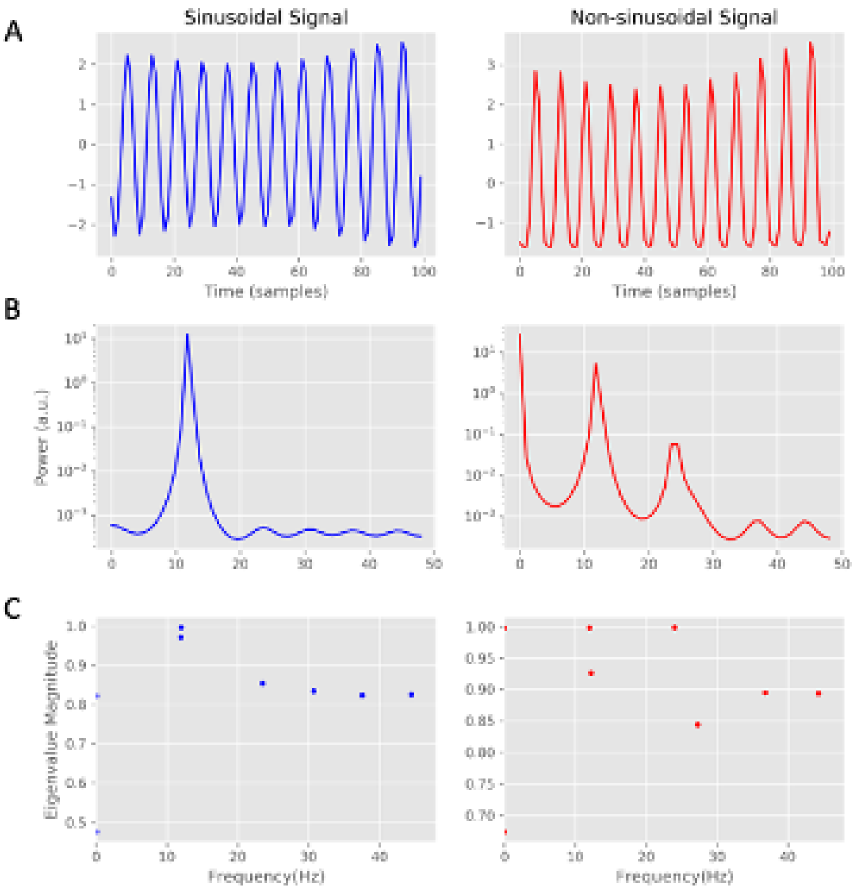

Figure G.13: A) a simulation containing a sinusoidal (left) and a non-sinusoidal oscillation (left). B) The power spectra of the two signals. The non-sinusoidal shape in the second oscillation induces a harmonic peak at 24Hz in the spectrum. C) the pole magnitude and peak frequency of the two signals. The harmonic peak is also represented by its own SSE mode. This might easily be mistaken for a separate oscillation rather than a harmonic of the 12Hz signal.

Appendix H. MVAR Hyper-parameter assessment

The MVAR model hyper-parameters of model order and sampling rate were assessed by a comparison across model orders 1, 4, 8, 12 and 16 alongside four different sampling rates. A MVAR model was fitted for each combination of parameters for one run from each participant (the full analysis used three runs per participant) and the average results are shown here. Figure H.14 shows the

Fourier power spectra for each combination of hyperparameters and figure H.15 shows the modal damping times and peak frequencies. The power spectra for the fastest sampling rates are dominated by the low-pass filter band. This is as the poles in a fitted MVAR model spread out to describe the largest spectral features across the whole zero to Nyquist frequency range. As a result, the poles of the modal decomposition cluster around the filter transitions rather than neuronally generated oscillatory peaks. At lower sampling rates, the effect of the filter is negligible and the mode poles are evenly distributed across frequency. In these sample rates any model order above 4 is able to resolve the alpha peak in the power spectrum. The final analysis was conducted using a sample rate of 120Hz and a model order of 12. This set of parameters provided a clear alpha peak without interference from the filter. The results in the bottom right of figures H.14 and H.15 also demonstrates that the core results are robust to moderate changes in these hyper-parameters. The power spectra and modes are very similar for a range of both model order and sample rate.

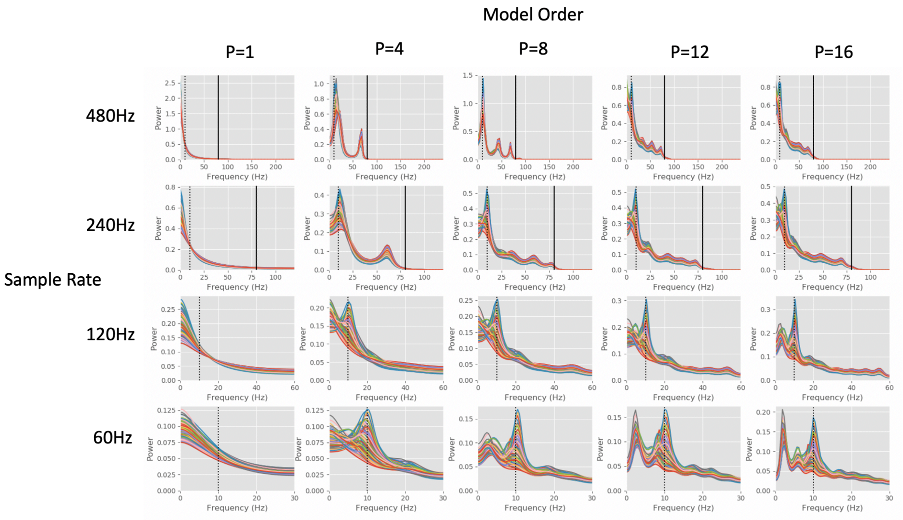

Figure H.14: Spectrum estimation from MVAR models computed across a range of model orders and sampling rates. A solid vertical line indicates the low-pass filter limit of 80Hz and the dotted vertical line indicates 10Hz in the alpha range.

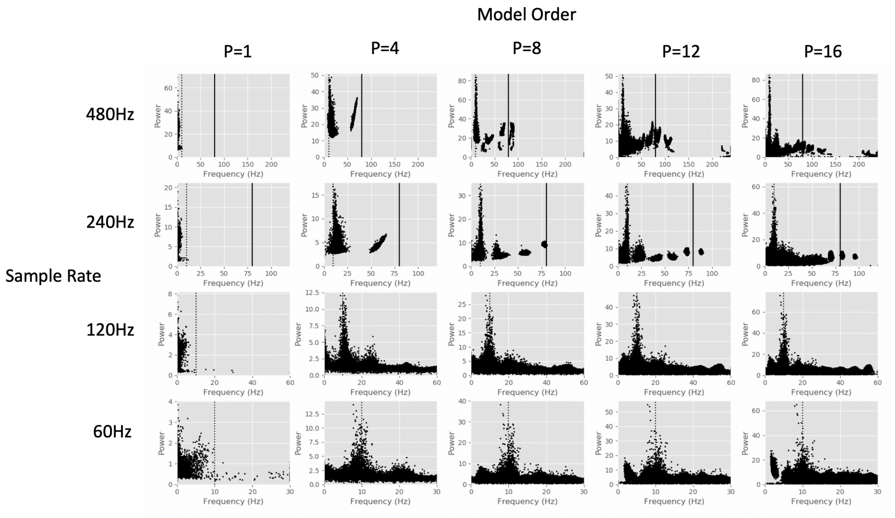

Figure H.15: Mode distributions from MVAR models computed across a range of model orders and sampling rates. A solid vertical line indicates the low-pass filter limit of 80Hz and the dotted vertical line indicates 10Hz in the alpha range.

Appendix I. Network eigenmodes structure

To further assess the more detailed network alpha network structure, we applied an eigenvalue decomposition to the group average alpha network matrix from Figure 4C. The results are shown in Figure I.16. The first two modes are subjectively similar to the components obtained from the PCA analysis in the main text. The first component captures the mean alpha power in occiptial cortex and the second captures and occipito-parietal gradient. The remaining modes capture more complex structure including a wider range of temporal and frontal regions, however in this instance these account for relatively little variance in the overall dataset. We believe that a wider, more complex range of spatial structures would be observed in task recordings rather than resting state. Similarly, more complex spectral structure would be observed in a temporally non-stationary analysis which are sensitive to transient bursts of power.

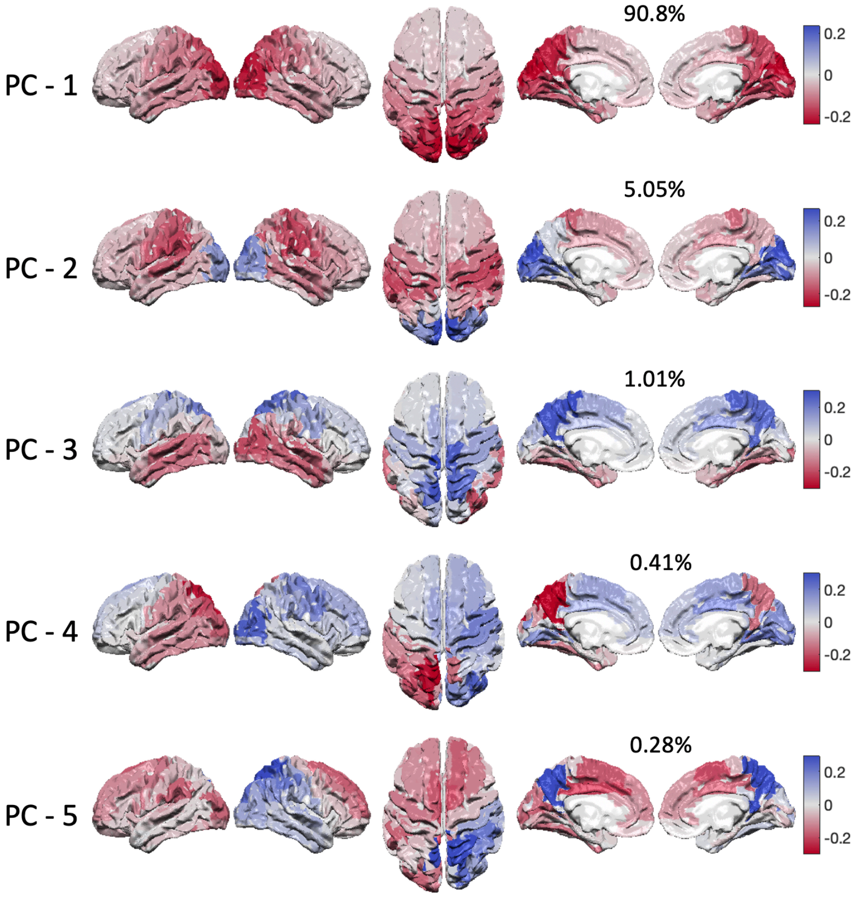

Figure I.16: The spatial structure and proportion of variance explained of the first five eigenvectors of the group average alpha network matrix.

Appendix J. Split-half reliability of alpha components

In Figure J.17 we show the split-half reliabilities of the 50 principal components of the PCA analysis in the alpha, theta and beta bands over 500 iterations of each. The first two components from the alpha band have a split-half correlation significantly above zero and are carried forward for detailed analysis.

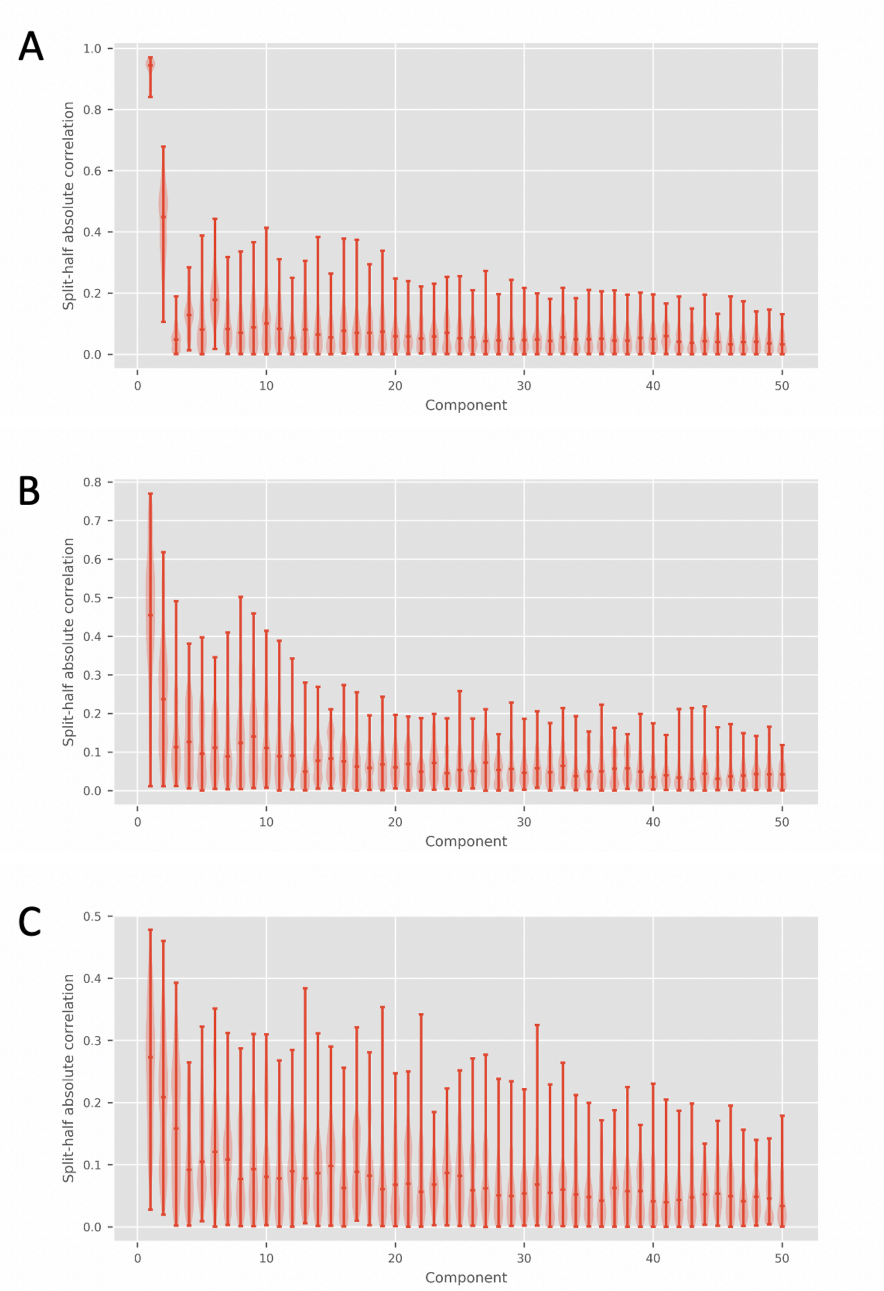

Figure J.17: Split-half reliability of the 50 principal components for the **A** alpha, **B** theta and **C** beta (bottom) SSEs. The split-half correlation values are shown as the distributions of the absolute value of the correlations (as the sign of the eigenvector-based map is confounded with the sign of the eigenvalue). The horizontal bar on each plot shows the median value.

Appendix K. Spatio-Spectral Eigenmodes in the theta and beta bands

Figure K.18 shows the surface power for the first four components of the theta and beta bands; this can be compared with figure 8 for the first two components of the alpha band analysis. Only the first two alpha modes were carried forward for further analysis, the spatial structure of the remaining modes are included here for reference.

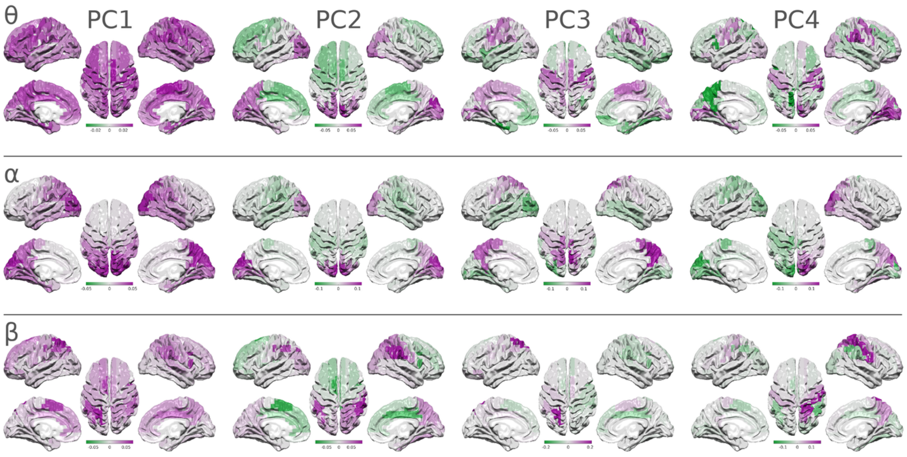

Figure K.18: Surface visualisations of the first four principal components for each of the theta, alpha and beta bands. These can be compared with the first two alpha band components shown in figure 8 of the main paper.

Appendix L. Relationship between PC-score and frequency for the alpha band.

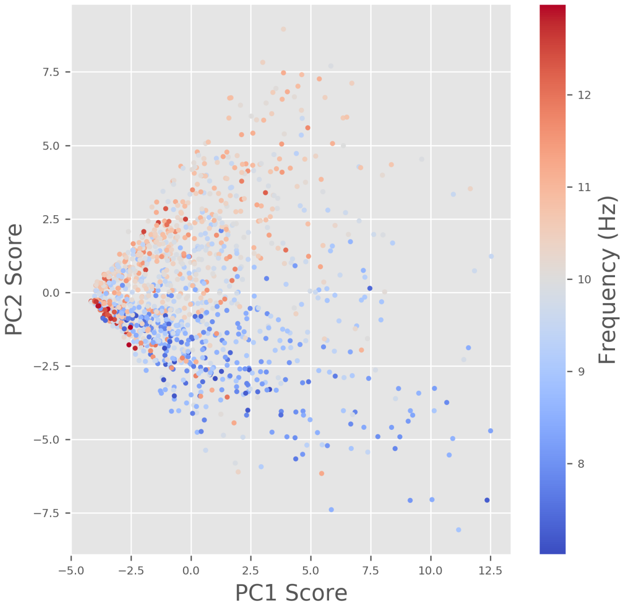

Figure L.19: Scatter plot of the PC-scores for the first two components of the results in the alpha band. Each point is a single SSE and colour indicates peak frequency. The x-axis contains the scores for PC-1 which correspond to overall alpha power. The PC scores are orthogonal and have no linear correlation. Low PC-1 scores indicate SSEs with low overall power whilst high scores indicate SSEs with strong alpha networks which, in turn, have greater variability in PC-2. The frequency correlation with PC2 can be seen as a greater density of high frequencies (red colours) above zero in the y-axis.

Appendix M. Effect of orthogonalisation.

The main HCP analysis pipeline was computed with and without symmetric orthogonalisation to assess the impact of this preprocessing stage. This analysis was carried out on one run from each participant, the full analysis used three

runs per participant. The group network structure is computed four times for each orthogonalisation condition. We compute the standard Fourier approach, the modal approach using all modes (equivalent to the Fourier approach), the modal approach using only significant modes and the modal approach using only residual modes. Figure M.20 shows the group average alpha band cross-spectral density matrices computed with and without orthogonalisation. A strong pattern of leakage can be see in the unorthogonalised networks using the Fourier, full-modal and residual modal approaches. This is strongly attenuated after orthogonalisation leaving a more physiologically plausible network structure. In contrast, the leakage pattern is not present in the significant modal approach either with or without orthogonalisation. The network structure in both cases is very close to the physiological network see after othogonalisation in the other approaches. This implies that source leakage is most strongly affecting the small, rapidly damped modes and that the strongly oscillatory modes carry similar information whether or not orthogonalization has been applied.

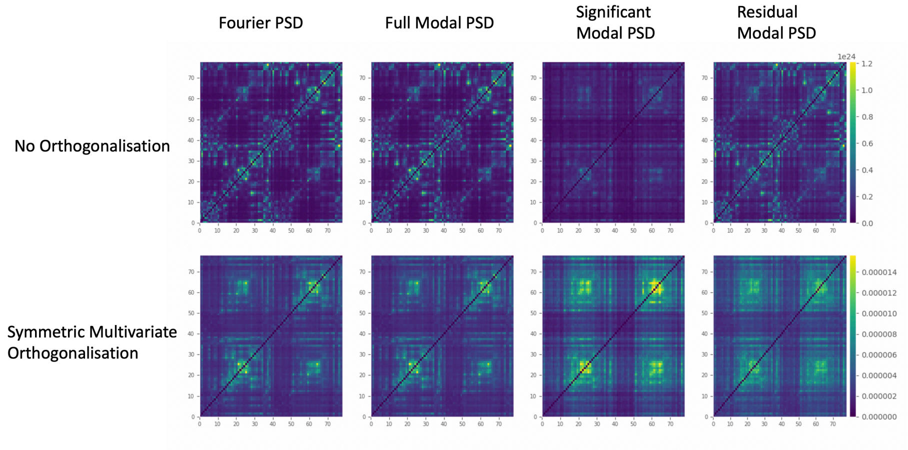

Figure M.20: The group-level cross-spectral density matrices between 7-13Hz computed with and without orthogonalisation.

The group average power spectra show some subtle differences between orthogonalisation conditions, reflecting differences in the spatial distribution of the oscillatory components(Figure M.21A). The distribution of poles across frequency and damping time is also very similar in both conditions, again indicating that the orthogonalisation is primarily impacting the spatial distribution of oscillations rather than their peak frequencies or dynamics (Figure M.21B). Finally, we repeat the PCA analysis in both conditions and show the scatter plot of the relationship between peak frequency and score for PC-2, the occipito-parietal mode identified in the main text (Figure M.21C). These results are similar in both conditions as the orthogonalisation has very little effect

on either the significant oscillatory modes or on the distribution of modes in frequency.

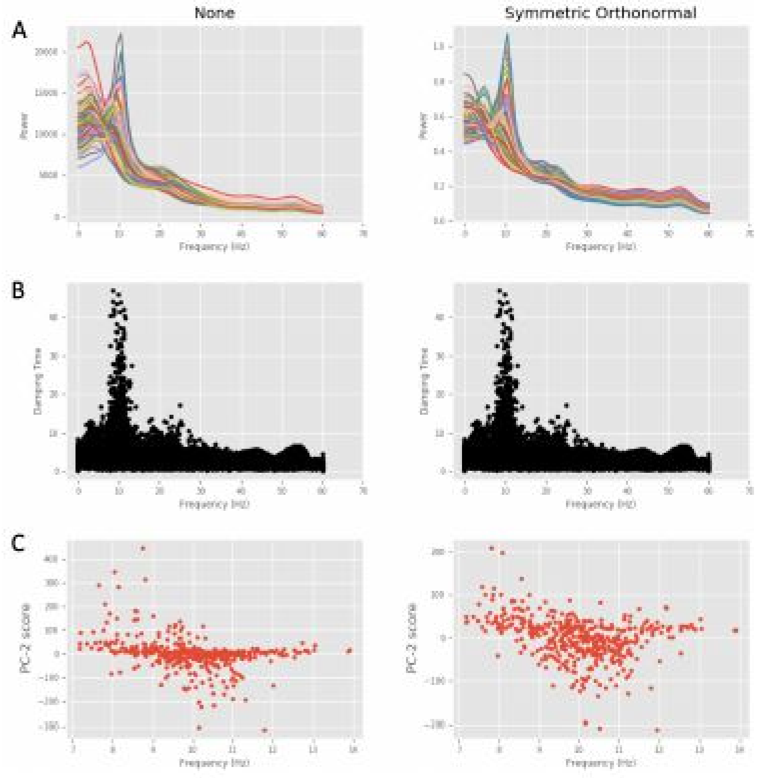

Figure M.21: A comparison of the power spectra and modal decompositions parameters with and without orthogonalisation.
A: Group average Fourier power spectra for each region in the parcellation.
B: The modal peak frequencies and damping times across the whole group.
C: Scatter plot of modal peak frequency against the component score for PC-2.
